# Dynamic properties of water in breast pathology depend on the histological compounds: distinguishing tissue malignancy by water diffusion coefficients

**DOI:** 10.1186/1756-0500-7-887

**Published:** 2014-12-08

**Authors:** Rustem F Baikeev, Roman A Gubanov, Kamil K Sadikov, Sufiya Z Safina, Farhat F Muhamadiev, Timur A Sibgatullin

**Affiliations:** Department of Biochemistry, Kazan State Medical University, Butlerova St., 49, Kazan, Tatarstan Russia; Kazan Oncological Dispensary, Baturina St., 7, Kazan, Tatarstan Russia; Kazan Institute of Biochemistry and Biophysics of Russian Academy of Science, Kazan, Tatarstan Russia

**Keywords:** Breast cancer, NMR, Self–diffusion coefficient, Morphology, H_2_O, Non-linear regression analysis

## Abstract

**Background:**

The parameters that characterize the intricate water diffusion in tumors may also reveal their distinct pathology. Specifically, characterization of breast cancer could be aided by diffusion magnetic resonance.

The present *in vitro* study aimed to discover connections between the NMR biexponential diffusion parameters [fast diffusion phase (D_FDP_ ), slow diffusion phase (D_SDP_ ), and spin population of fast diffusion phase (P_1_)] and the histological constituents of nonmalignant (control) and malignant human breast tissue. It also investigates whether the diffusion coefficients indicate tissue status.

**Methods:**

Post-surgical specimens of control (mastopathy and peritumoral tissues) and malignant human breast tissue were placed in an NMR spectrometer and diffusion sequences were applied. The resulting decay curves were analyzed by a biexponential model, and slow and fast diffusion parameters as well as percentage signal were identified. The same samples were also histologically examined and their percentage composition of several tissue constituents were measured: parenchyma (P), stroma (St), adipose tissue (AT), vessels (V) , pericellular edema (PCE), and perivascular edema (PVE). Correlations between the biexponential model parameters and tissue types were evaluated for different specimens. The effects of tissue composition on the biexponential model parameters, and the effects of histological and model parameters on cancer probability, were determined by non-linear regression.

**Results:**

Meaningful relationships were found among the *in vitro* data. The dynamic parameters of water in breast tissue are stipulated by the histological constituents of the tissues (P, St, AT, PCE, and V). High coefficients of determination (R^2^) were obtained in the non-linear regression analysis: D_FDP_ (R^2^ = 0.92), D_SDP_ (R^2^ = 0.81), and P_1_(R^2^ = 0.93).

In the cancer probability analysis, the informative value (R^2^) of the obtained equations of cancer probability in distinguishing tissue malignancy depended on the parameters input to the model. In order of increasing value, these equations were: cancer probability (P, St, AT, PCE, V) (R^2^ = 0.66), cancer probability (D_FDP_, D_SDP_)(R^2^ = 0.69), cancer probability (D_FDP_, D_SDP_, P_1_) (R^2^ = 0.85).

**Conclusion:**

Histological tissue components are related to the diffusion biexponential model parameters. From these parameters, the relative probability of cancer in a given specimen can be determined with some certainty.

## Background

Cancer diagnoses proceed in several steps, each with varying reliability (%): (1) Revelation of paraneoplastic clinical syndromes (30 − 40%), (2) Positive values of laboratory immunochemical markers (30− 40%; 75 − 84% in advanced cases), and (3) Histological revelation (95 − 97%). Histological diagnosis is based on appearance of atypical cells and tissues, amount of mitosis, the state of the tumor’s boundaries and surrounding tissues and whether the tumor has invaded the vessels through the basal membrane − cancer *in situ*.

Non-invasive (objective) detection and diagnosis of breast cancer is essential for successful treatment. Magnetic resonance (MR) has become an increasingly popular technique for detecting and delineating breast cancer in everyday practice.

MR theory attempts to relate MR signal parameters to the microstructural and physiological features of tissues, enabling a non-invasive nosological diagnosis, especially of cancer.

Diffusion (self-diffusion) is the process by which molecules or ions are randomly shifted (Brownian motion) under the action of internal thermal energy. Intracellular water exists in both “free” and “bound” states, which are easily distinguished by the time of NMR–^1^H (T_1_ and T_2_) relaxation. Aqueous ion, protein, lipid and nucleotides systems are known to hold “hydration water” at their interfaces. Unlike regular water, which freezes around 0°C, hydration water remains fluid down to ~200 K (−73°C). ^17^O data show that hydration water is less mobile than free water and undergoes anisotropic motions [1].

Self-diffusion between the two water phases (two domains) can occur by molecular transfer, or may be triggered by the pH conditions. In the latter case, the mean residence time of a water molecule is of the order of 10^−3^ s at room temperature and pH 7 [2]. Intracellular water moves chiefly by Brownian displacement; cytoplasmic streaming plays at most a minimal role [3].

Nuclear Magnetic Resonance (NMR) is useful for studying the static properties of matter (i.e. structure) and its dynamic properties such as self-diffusion, flow and relaxation.

The pulsed field gradient (PFG) NMR method, pioneered by Stejskal and Tanner [4], remains one of the main techniques for obtaining dynamic information such as self-diffusion coefficients.

The displacement sensitivity of PFG NMR is approximately 100 nm and diffusion coefficients can be measured down to approximately 10^−14^ m^2^ s^−1^[5]. Therefore, PFG NMR is an excellent tool for probing molecular diffusion and structure in biological systems, and is especially convenient because it requires no labeled probe molecules. The theory behind the PFG method has been well-developed [6]. Briefly, the Hahn spin-echo pulse sequence is modified into a PFG spin-echo pulse sequence, in which each period (τ) is spiked with a “rectangular” magnetic field gradient pulse of duration δ and magnitude g. The separation t_d_ between the leading edges of the gradient pulses specifies the time over which diffusion is measured. If the spin moves along the direction of the field gradient during t_d_, the phase change induced by the first gradient pulse is not cancelled by the phase change induced by the second identical gradient pulse. Averaged over a spin ensemble, this phase shifting effect diminishes the signal. The extent of diminution is proportional to the net displacement of the spin along the direction of the gradient during t_d_. The apparent diffusion coefficient (ADC), which need not equal the true coefficient, is frequently determined from the initial slope of the attenuation plot. Variations in the experimental conditions are usually quantified by the value of b = γ^2^δ^2^g^2^t_d_.

In studies of water diffusion in biological systems, any specific NMR attenuation curve may be modelled by a broad range of mathematical functions: biexponential, multiexponential and nonexponential.

Scientists have long sought the physical cause of the biexponentiality of the diffusion signal decay function. The problem of self-diffusion coefficient measurements during interphase exchange was first resolved by Kärger [7]. They assumed a biphasic system in which the exponential function distribution depends on the lifetime of the kinetic unit in the two phases. They fitted the diffusion decay A(t_d_) record as a function of t_d_. However, this model does not account for the restricted diffusion through biological membranes (cell and organelle boundaries), or the relaxation time difference between the two domains. These limitations have been discussed in relation to diffusion in the brain [8]. Price modified [9] Kärger’s model to accurately quantify water diffusion, but this model is limited to spherical interfaces such as isolated human breast cancer cells in culture [10].

Models based on dynamic parameters (such as membrane restriction and permeability) [11] and geometrical features (such as planes and cylinders) [12] have also been proposed. In all of these diverse models, the diffusion signal decay is well-approximated by a biexponential function [13].

Currently, researchers accept two ways of describing NMR diffusion decay in complex biomedical samples and tissues, even when the detailed morphology of the sample is unknown.

The first approach considers tissue as a simple bicompartmental model comprising extracellular and intracellular spaces. In this model, the apparent diffusion coefficient (ADC_m_) is obtained from the volume-weighted quantities V_SDP_ (the intracellular slow diffusion phase (SDP) of water) and V_FDP_ (the extracellular fast diffusion phase (FDP) of water), and the *average* intracellular and extracellular diffusion coefficients (D_SDP_ and D _FDP_, respectively) in slow exchange [14]. The ADC is then computed as ADC_m_ = (V_SDP_D_SDP_ + V_FDP_D_FDP_)/(V_SDP_ + V_FDP_) (1).

The second approach is based on diffusion NMR observations; namely, that diffusion in biological tissues is well-fitted to a biexponential function corresponding to a slow diffusion phase (SDP) and a fast diffusion phase (FDP) in slow exchange: S = S_0_P_1_ exp(−bD_FDP_) + S_0_P_2_ exp(−b_SDP_) (2) [15]. Here, S is the MRI signal at a particular **b** value, S_0_ is the signal at **b** = 0, and D_FDP_ and D_SDP_ are the diffusion coefficients in the fast and slow diffusion phases respectively, with P_1(FDP)_ + P_2(SDP)_ = 1.

In fact, the estimated diffusion coefficients and volume fractions of the SDP and FDP have been strikingly consistent across the literature [10, 16–18].

Based on the data accumulated in NMR studies, scientists have quantitatively differentiated malignant tissues by evaluating their diffusion coefficients [19]. DW (diffusion-weighted) MRI provides significant opportunities for accurately assessing how breast cancer patients respond to neoadjuvant chemotherapy at an early stage, since it enables voxel-based image analysis [20]. Consequently, evaluating the dynamic state of water in cancerous breast tissues is important for determining the degree of a neoplasm process. Thus, the MRI values related to tumor cellularity can be used to differentiate malignant breast lesions from benign ones.

A mean diffusivity (MD) threshold of 1.1 × 10^−9^ m^2^/s discriminates malignant from benign breast lesions with a specificity and sensitivity of 81% and 80%, respectively [21]. In the same study, a cut-off of 1.31 × 10^−9^ m^2^/s (MD of malignant lesions −2 SD) reduced the specificity to 67%, but achieved 100% sensitivity [21]. The cut-off requirement [22] is a distinct disadvantage of this approach in cancer diagnosis, since it is relative and depends on the biochemical constituents of the patient’s own tissues [23]. These constituents influence the morphology and anisotropic diffusion properties of breast tissues [24, 25]. The MR scanner system [21, 26], magnetic field strength [21, 27, 28], acquisition sequence [19, 26], b-value [27, 29, 30], fat suppression method [31] should also be considered.

The present *in vitro* study aimed to discover connections between the NMR biexponential diffusion parameters and the histological constituents of the nonmalignant (control) and malignant human breast tissues. It also seeks to distinguish breast tissue status from the measured water diffusion coefficients.

## Methods

Seventeen female patients with breast pathology were recruited for this study; six control subjects (mastopathy, peritumoral areas) and eleven breast cancer patients (T_2_N_0_M_0_, *n* =6; T_2_N_1_M_0_, *n* =5). The mean age of the cancer patients was (59 ± 4) years, and mean cancer duration was 65 days (range 10–125 days). The inflammatory symptoms were as follows: pain (3 patients), erythema (2 patients), heat (1 patient); one patient reported a family history of breast cancer. Single specimens (≈1 cm × 0.5 cm × 0.5 сm, ≈0.25 gram) were excised from the operation tissue of each patient and preserved for examination (*n* =17). Samples were maintained at 275–277 K (2–4°C) until required. Abundance of fat was an exclusion criterion for specimens.

**Figure 1:**
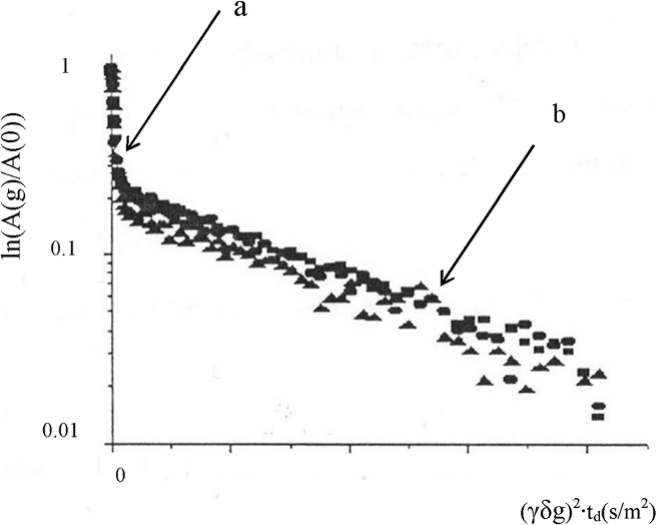
**Scheme of the slope of function A(g) at the different time of diffusion: t**_**d1**_ **(■) <t**_**d2**_ **(**  **) <t**_**d3**_ **(▲). a**- fast diffusion phase, **b**- slow diffusion phase.

NMR examination was performed *in vitro* two hours after the operation, applying the “stimulated echo” method [32], without spinning of the specimens. The self-diffusion and relaxation times (T_1_ and T_2_) of water and organic compounds in the tissues were determined by a ^1^H–NMR-analyzer “Spin Track” (Resonance Systems Ltd., Yoshkar-Ola, Russia) operating at 19.1 MHz and equipped with the electromagnet. The maximal amplitude of the magnetic field gradient pulse g was set to 4 T(Tesla)/m. The diffusion attenuation of the spin echo signal, that is, the dependence of the echo amplitude A(g) on the gradient pulse amplitude in the coordinates, is revealed in the ln [A(g)/ A(0)] versus b plot presented in Figure 1, where b = γ^2^δ^2^g^2^t_d_ (the diffusion time t_d_ is 20 ms), A(0) is the echo amplitude in the absence of a magnetic field gradient, g_0_ = 1 × 10^−3^ T · m^−1^, γ is the gyromagnetic ratio for protons, and δ =0.2 ms is the pulse duration; 2τ =20 ms, π/2 = 8 μs. The repetition time is 2 s. The mean ADC (ADC_m_) depends on both D_FDP_ and D_SDP_, which quantity was investigated to enable comparison of our data with the results of *in vivo* experiments and the ADC_m_ values among different sample groups. The ADC_m_ was determined from the initial slope of the attenuation plot A(g) (Figure 1) as ADC_m_ = −1/t_d_ • (∂ln[A(g)/A(0)]/∂( γδg)^2^) | _γδg→0_. The slope of the function A(g) was approximated by a programmed version of the “peel-off” method [33]. In this presentation, the self-diffusion coefficients (D _FDP_ and D_SDP_) are determined by the tangent to the angle of the exponential decay curves of the function A (g) (Figure 1).

Under the experimental conditions of the present study, diffusion was derived from the apparent (not the true) values of ADC_m_, D_FDP_ and D_SDP_. The dependence of ADC on t_d_ (*n* = 3) in the peritumoral breast tissue specimens was examined separately, with t_d_ = 11, 50, 200, or 800 ms.

Measurements were performed at (310 ± 0.2) K [(37 ± 0.2)°C]. A water (H_2_O; Sigma-Aldrich Corp., St. Louis, MO, USA) sample was used as a standard. At 303 K (30°C), water attenuation was described by a single exponential diffusion decay; namely, by A(g)/A(0) = exp(−kD), where D is independent of t_d_ and equal to 2.7 × 10^−9^ m^2^/s.

Once the NMR studies were complete, the breast tissue specimens were immersed in formaldehyde and their histology was examined. The time elapsed between tissue excision during the operation and fixation of the specimens was approximately 2 hours. The percentages of relevant histological constituents, namely, parenchyma (P), stroma (St), adipose tissue (AT), vessels (V), pericellular edema (PCE), and perivascular edema (PVE), were calculated by the “net of random step” method [34] and measured as relative units (%). Different groups of tissue specimens were statistically compared by the Wilcoxon rank-sum test [35]. Correlation (Spearman’s coefficient) and multiple non-liner regression analyses were performed using Statgraphics Centurion XV Professional (USA). Data were fitted to a second-order regression equation [35]:



This study was approved by the local ethics committee of Kazan State Medical University (protocol No.3, 04.04.2012). Written informed consent was obtained from all participants prior to the study.

## Results and discussion

### Morphology: agreement between *in vivo*and *in vitro*NMR studies

Analyses were conducted according to the TNM classification and stroma contents (Tables 1 and 2). Female patients at tumor stage T_2A_ or T_2B_ only were selected for the study, for the following reasons: (1) In the Republic of Tatarstan (Russia) 63.33% of women undergoing treatment in oncological clinics are hospitalized at stage T_2_N_(0,1,2)_M_0_[36]; (2) Restricting the tumor stage ensured a homogeneous cohort for the study.

**Table 1:** **Morphometry features (share,%) of the mammary gland tissue samples**

Diagnosis	Parenchyma	Stroma	Adipose tissue	PCE	PVE	Vessels
1. Control n = 6	1.1.	1.2.	1.3.	1.4.	1.5.	1.6.
	16,7 ± 19,7^1^	35,7 ± 8,8	32,6 ± 21,0	4,0 ± 6,4	5,9 ± 5,8	5,1 ± 4,9
	(0,01 - 54,7)^2^	(25,5-49,0)	(0,01-53,8)	(0,01-17,0)	(0,01-13,6)	(0,2-14,1)
	5470^3^	1,9	5380	1700	1360	70,5
2. Breast cancer n = 11	2.1.	2.2.	2.3.	2.4.	2.5.	2.6.
	30,7 ± 15,7	39,9 ± 13,6	11,6 ± 16,1	7,1 ± 6,9	7,5 ± 11,1	3,1 ± 2,6
	(10,3-56,6)	(26,1-64,2)	(0,01-50,4)	(0,01-16,0)	(0,01-32,3)	(0,1-7,3)
	5,5	2,5	5040	1600	3230	73

**Note:** n - number of the samples; ^1^ – mean value ± SD, ^2^ - range of parameter; ^3^ - ratio of high/low range values of the certain morphological constituents percentage.

Comparison of 2 groups (Wilcoxon): 1.1-2.1 p < 0,028 1.4-2.4 p < 0,047.

1.2-2.2 p > 0,6 1.5-2.5 p > 0,17.

1.3-2.3 p < 0,047 1.6-2.6 p < 0,047.

**Table 2:** **Self-diffusion coefficients of the water molecules in breast cancer**

Parameter	Control n = 6	Breast cancer			Share of stroma ^*^ (%) (control + cancer)	
		The entire group of cancer specimens n = 11	T _2_ N _0_ M _0_ n = 6	T _2_ N _1_ M _0_ n = 5	< 50 n = 14	≥50 n = 3
	1	2	3	4	5	6
ADC_m_ · 10^−9^ (m^2^/s)	0,78 ± 0,28^1^	1,62 ± 1,28	0,85 ± 0,34	2,54 ± 1,30	0,91 ± 0,28	3,25 ± 0,75
	(0,41-1,16)^2^	(0,33-3,43)	(0,33-1,22)	(0,79-3,43)	(0,41-1,35)	(0,33-3,43)
	2,84^3^	10,39	3,69	4,34	3,30	10,39
D_FDP_ · 10^−9^ (m^2^/s)	1,25 ± 0,13	0,97 ± 0,25	0,93 ± 0,24	1,01 ± 0,28	1,06 ± 0,27	1,10 ± 0,18
	(1,10-1,40)	(0,57-1,25)	(0,60-1,20)	(0,57-1,29)	(0,57-1,40)	(0,90-1,20)
	1,27	2,19	2,00	2,25	2,46	1,34
P_1 (share)_	0,48 ± 0,17	0,75 ± 0,22	0,74 ± 0,28	0,78 ± 0,14	0,69 ± 0,24	0,53 ± 0,26
	(0,27-0,77)	(0,23-0,97)	(0,23-0,97)	(0,63-0,95)	(0,27-0,97)	(0,23-0,72)
	2,84	4,23	4,22	1,51	3,59	3,14
D_SDP_ · 10^−11^ (m^2^/s)	1,67 ± 0,15	0,86 ± 0,68	1,75 ± 0,17	2,00 ± 1,03	1,69 ± 0,16	2,30 ± 1,34
	(0,50-1,91)	(0,40-3,84)	(1,50-1,98)	(1,40-3,84)	(1,40-1,98)	(1,50-3,84)
	3,82	9,6	1,32	2,74	1,41	2,56

**Note:** n - number of the samples; ^1^ – mean value ± SD, ^2^ - range of parameter.; ^3^ - ratio of high/low range values of the certain diffusion parameter.

*- crosslinked collagen is embedded into the carbohydrate matrix of stroma.

Comparison of 2 groups (Wilcoxon):

ADC_m_: 1-2 p<0,03; D_FDP_: 1-2 p<0,05; P_1_: 1-2 p>0,15; D_SDP_: 1-2 p>0,9; 1-3 p>0,9; 1-3 p<0,05; 1-3 p>0,15; 1-3 p>0,3; 1-4 p>0,7; 1-4 p>0,05; 1-4 p<0,05; 1-4 p=1; 1-5 p>0,7; 1-5 p>0,5; 1-5 p>0,05; 1-5 p=1; 1-6 p=1; 1-6 p>0,5; 1-6 p>0,2; 1-6 p=1; 3-4 p>0,2; 3-4 p>0,8; 3-4 p>0,8; 3-4 p>0,5; 5-6 p=1; 5-6 p>0,5; 5-6 p>0,1; 5-6 p=1.

**Figure 2:**
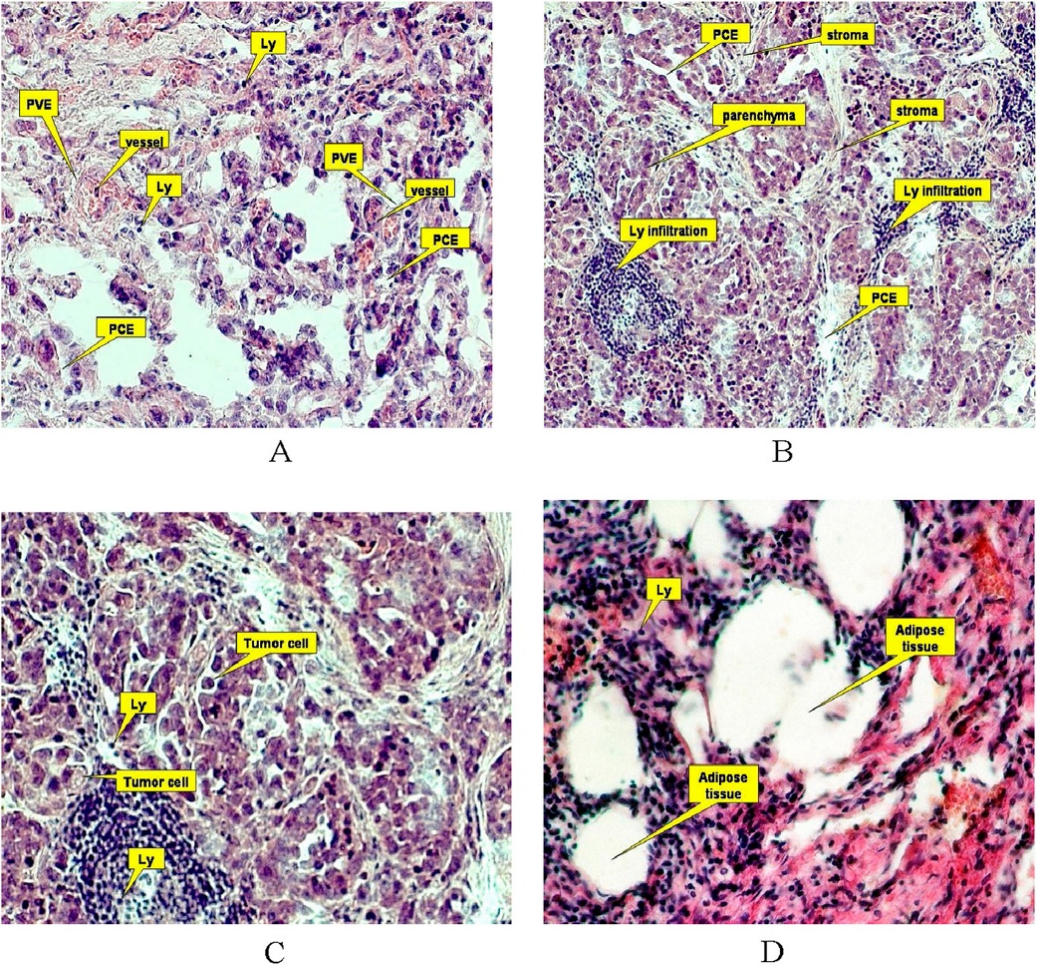
**Breast cancer morphology (A-D).** Ly-lymphocyte (lymhocyte size≈7 μm) is used as a unit; PVE - perivascular edema; PCE - pericellular edema; C – tumor cell enriched in vacuoles is visible on the left side of the microscopic field.

Instances of mastopathy and fibroadenoma in the control group were of the pericanalicular type, with concentric proliferation of the intralobular connecting tissue around channels. Tissue anomalies were restricted in size. Some of the cancer specimens were scirrhous, and hyalinization of the connecting tissue impregnated with small groups of tumorous cells was observed. In most cases, adenocarcinoma was classified among the infiltrative carcinomas. Tubular or solid glandular-like structures were located in the thick connecting tissue (Figure 2, A–D). The P, AT, PCE and V parameters were significantly different (*p* <0.05) among different groups (Table 1).

Our analysis first investigates whether the results of the *in vitro* experiments accord with those obtained *in vivo*. NMR measurements *in vivo* and *in vitro* reflect the vitality and proton relaxation characteristics of the tissues [2, 37]. Nevertheless, *in vitro* diffusion studies are important because they provide biochemical and biophysical information that reflects the state of malignant tissues. By contrast, because *in vivo* diffusion studies average the ADC, they exclude information on the FDP and SDPs of water (diffusion coefficients and spin populations) [38–40].

The absolute ADC values (in units of 10^−9^ m^2^/s) obtained *in vivo* for malignant mass have been reported as 0.95 ± 0.18 [21], 0.97 ± 0.20 [19], 0.99 ± 0.18 [27], 1.03 ± 0.02 [28], 1.021 [26], 1.17 ± 0.24 [41], and 1.22 ± 0.31 [42]). The ADC values of benign masses were reported as 1.47 ± 0.21 [27], 1.48 ± 0.37 [21], 1.488 [26], 1.57 ± 0.23 [19] and 1.67 ± 0.54 [42]), while those of cysts were 2.25 ± 0.26 [21] and 2.64 [27], and those of normal tissues – were 1.85 ± 0.22 [27] and 2.09 ± 0.27 [42]. These ranges include the *in vitro* ADC_m_s reported in Table 2.

Among the wide ADC range reported in the literature [(0.664–1.359) × 10^−9^ m^2^/s] [43], 30% and 70% of breast cancer lesions were characterized by ADC >1.44 × 10^−9^ m^2^/s and ADC ≤1.44 × 10^−9^ m^2^/s, respectively [44]. Our data (Table 2) reveal the cause of this discrepancy. We found that most of the biological tissue parameters are non-parametric; that is, they cannot be approximated by a standard data distribution function (such as normal, Gaussian, exponential). Consequently, their standard deviation is large relative to the mean. Second, breast tissues are characterized by high morphological spatial heterogeneity (Table 1; Figure 2).

The current literature establishes no reliable ranges of the absolute ADCs of breast cancer tissues. The ADC of pure mucinous breast carcinoma is (1.8 ± 0.4) × 10^−9^ m^2^/s [45]. Compared with inflammatory breast diseases, the ADC in breast cancers is lower at the wall (1.09 × 10^−9^ m^2^/s vs. 1.42 × 10^−9^ m^2^/s) and higher in the central region of the tumor (1.94 × 10^−9^ m^2^/s vs. 1.05 × 10^−9^ m^2^/s). In the central region of an invasive ductal carcinoma, the ADC was reported as 2.7 × 10^−9^ m^2^/s [38].

### Diffusion coefficients reflect the compartmentalization of water in tissues

MR studies of intracellular water generally require that the intracellular and extracellular water signals be clearly distinguished.

The difference between the intracellular and extracellular water states in the NMR-^1^H range of the spin-echo decay structure is complicated, but may be described by the biphasic diffusion model with interface exchange (see Eq. (2) [15] in the background, Figure 1 and Table 3).

**Table 3:** **Self-diffusion coefficients of molecules in the peritumoral breast tissue (n = 3)**

№	Registered parameter	Time of diffusion (t _d_ , ms)			
		11	50	200	800
1	ADC_m_ (10^−9^ m^2^/s)	0,73 ± 0,55	0,75 ± 0,05	1,0 ± 0,1	1,4 ± 0,1
2	D_FDP_ (10^−9^ m^2^/s),	1,6 ± 0,1	1,4 ± 0,1	1,7 ± 0,2	1,5 ± 0,2
3	D_SDP_ (10^−11^ m^2^/s)	2,2 ± 0,1	2,0 ± 0,1	1,8 ± 0,2	2,2 ± 0,3
4	Р_1_ (share)	0,49 ± 0,01	0,52 ± 0,01	0,70 ± 0.01	0,90 ± 0.01

Note: n – number of the specimens.

The FDP and SDP volume fractions (70% and 30% respectively; see Table 2, P_1_ = 48–75%) disagree with the volume fractions of the extra- and intracellular compartments [46]. Therefore, the FDP and the SDP cannot be directly assigned to these physical compartments. However, experimental evidence exists that the volume variations of the SDP and FDP highly correlate with the volume variations of the intra- and extracellular spaces as cells enlarge or shrink under different physiological, pathological or experimental conditions [47, 48].

This mismatch could be partially attributable to the extracellular space occupied by various structures, which mimics the intracellular space at the NMR scale. Likely contributors are sclerotic tissue, matured stroma - crosslinked collagen embedded into the carbohydrate matrix, and fat aggregates. Сollagen becomes crosslinked when some of the lysyl and hydroxy lysyl side-chains of the amino groups bond to aldehyde groups under the action of a copper-containing oxidase [49, 50]).

The local extent of stroma may exceed 200 μm (Figure 2A), and fat deposition covers several hundred micrometers (Figure 2D). Cancerous tissues are likely to be affected by additional structures. Because the permeability of blood vessels is increased in cancer tissue, fibrin is deposited in the interstitial spaces [51], followed by calcium hydroxylapatite (Ca_10_(PO_4_)_6_^.^(OH)_2_) deposition. The space occupied by these structures may be misinterpreted as intracellular space, leading to overestimates. Vacuoles and vacuole-like structures (Figure 2C) are regarded as intracellular spaces [52] and therefore part of the FDP. Although intracellular water has a low diffusion coefficient (ranging from 0.3 × 10^−9^ m^2^/s to 0.4 × 10^−9^), the diffusion coefficient of extracellular water approaches that of pure water ((3.0–3.25) × 10^−9^ m^2^/s at 310 K (37°C)) [11]. This wide disparity cannot be resolved by diffusion coefficient measurements.

Because free water can rapidly diffuse through intracellular material (at up to two thirds the rate of pure water) [53], the intracellular space may mimic the extracellular space; consequently, the two spaces are indistinguishable by this parameter.

The intracellular space of some cells yields both fast and slow water ADC components. These arise from the cytoplasm [FDP = (0.48 ± 0.14) × 10^−9^ m^2^/s; SDP = 0,034 × 10^−9^ m^2^/s] and the nucleus [FDP = (1.31 ± 0.32) × 10^−9^ m^2^/s; SDP = (0.057 ± 0.073) × 10^−9^ m^2^/s] [46].

The intracellular NMR water signal can be monitored in several ways; ct monitoring (by constant diffusion time experiment), ss monitoring (in which a single signal is monitored at large b value), and cg monitoring (by constant gradient experiment). In a ct experiment on perfused F98 glioma cells at small b, the signal was induced by extracellular and free diffusing water, and the ADC^ct^ was reported as (3.7 ± 0.2) × 10^−9^ m^2^/s. At larger b values the attenuation slope rapidly decreases to ADC^ct^ = (6.0 ± 0.002) × 10^−11^ m^2^/s. The low ADC^ct^ manifests from the restricted diffusion of water inside cells, which suggests that intracellular signals are separable from their extracellular counterparts, and that intracellular signals can be separately detected when b is large [48].

Water diffusion measurements conducted at extremely high b values revealed a multi-exponential decay of the water signal. This indicates the presence of two or three ADCs, depending on the range of the b-value. Changes in the intracellular signal component have been used to probe the intracellular volume and exchange time under various cellular constraints, such as osmotic stress, apoptotic conditions, immunosuppressive stress, and mercury reagents. In a cg experiment, the mean intracellular residence time of water was determined as approximately 50 ms [48].

Studies of several cell types have indicated three diffusion characteristics of intracellular water that clearly distinguish it from freely diffusing water (i.e., pure liquid water or dilute aqueous solution). In particular, (1) At typical diffusion times of MR experiments (1–100 ms), the apparent diffusion coefficient (ADC) of intracellular water is less than that of pure water at the same temperature; (2) The ADC of intracellular water decreases with increasing diffusion time; (3) For intracellular water, the MR diffusion signal, denoting the echo amplitude profile of the pulsed field gradient (PFG) acquired during a fixed diffusion time, frequently decays as a non- or monoexponential function of the diffusion-weighting b value [48]. These characteristics imply that intracellular water diffusion cannot be characterized by a single ADC. Water diffusion in the cytoplasm of isolated cells, comprising part of the intracellular space, also supports a multiexponential model [46].

In the high b_i_ range (5900–7800 s/mm^2^), the signal from fast-moving water is negligible [47]. The extracellular water signal can be suppressed by applying a slice-selective spin-echo pulse sequence combined with fast-flowing perfusion media [54], ensuring that the MR signal arises only from intracellular water [53].

Although these experiments certainly separate different water populations, the correlation between these populations and the known physiological compartments is less straightforward.

Depending on the study objectives and experimental conditions, diffusion decay may follow a biexponential, triexponential [55], multiexponential or nonexponential trend. Furthermore, neither intracellular nor extracellular water diffusion can be characterized by a single ADC. The FDP and SDP can be precisely assigned to extracellular (V_ex_) and intracellular (V_in_) portions only in packed cells, cultured cells [53], and some isolated tissue structures, such as neurons and muscle strips.

Under the experimental conditions of this study, the A(t_d_) with exchange and restricted diffusion is described by a simple sum of two exponents (see Eq. (2) [15] in the background). Recall that we have used the apparent (not the true) values of P_1_, P_2_, D_FDP_, D_SDP_ (Tables 2 and 3). The true values of these parameters are their limit values as 2τ → 0.

Therefore, the obtained D_FDP_ and D_SDP_ values (Table 2) compartmentalize the water in breast tissues based on the dynamic properties of water, which may not match the histological location. The latter must be elucidated in 2D and 3D diffusion measurements at the microscopic scale of NMR.

### Time-dependent diffusion coefficient and its relationship to tissue and medium geometry

#### Short- and long-time diffusion measurements

The time dependence of diffusion coefficients was initially studied in periodic arrays of parallel non-biological membranes [56]. However, this study overlooked the decrease of the diffusion coefficient at the membrane relative to its bulk value. Instead, it assumed a universal  behavior dependent only on the surface-to-volume ratio (SV) of the membranes.

The time-dependent diffusion of water and solvents in porous and semipermeable structures has been used to estimate the porous surface and its volume ratio [57], thereby obtaining the average and effective pore size [58] and the deviation of the pore from a spherical geometry [59]. Time-dependent diffusion coefficients in porous media with piecewise-smooth pore-grain interfaces have been evaluated [60] at short times (<2 ms) and may be simultaneously used to determine S/V (where S is the surface area, V is the pore volume).

The self-diffusion of polymer (polyethylene glycol and dextran) in cartilage largely depends on the observation time; short-time self-diffusion coefficients (diffusion time t_d_ ≈ 15 ms) are influenced by a strong non-specific obstruction effect imposed chiefly by the molecular weight of the polymers and the water content of the cartilage. More specifically, the measured self-diffusion coefficients decrease as the molecular weight of the polymers increases, and as the water content of the cartilage decreases. In contrast, the long-time self-diffusion coefficients of polymers in cartilage (diffusion time td ≈ 600 ms) reflect the structural properties of the tissue [61].

To obtain the V/S ratio, the short slope of the Padé approximant was fitted by the equation [60] (where *D*_0_ is the bulk diffusion coefficient of the fluid and *D*(t) is a time-dependent ADC). This result favorably agrees with the size obtained by microscopy [62]. Later, this approach was used to combine PFG with the gradient/radio frequency pulse sequence, yielding several parameters of biological cells; namely, the diffusion coefficient of free intracellular water, the surface-to-volume ratio, the average cell radius, and the variance of cell radius in a collection of cells [53].

To elucidate the restrictions imposed on translational motions of liquid molecules in cells, we investigated the effect of t_d_ on D_i_ in human breast tissues (Table 3).

Two of the diffusion coefficients were independent of diffusion times ranging from 11 ms to 50 ms. This implies that exchange between the two phases occurs on a much slower timescale (5 · 10^−2^ s) [63].

The absolute values of D_FDP_ and D_SDP_ were independent of t_d_ throughout the studied range (Table 3). Time-independence of the measured diffusion constant has sometimes been attributed to unrestricted diffusion [64]; however, it may also be an artefact arising from probing times that are much longer than the time of the restrictive effect [62]. Tissue geometry should be analyzed at short diffusion times and gradient pulses (i.e. path lengths shorter than the unit length of the structure) [32]. On larger scales, only the fully restricted (or averaged) diffusion constant is obtained, which equals the asymptotic diffusion coefficient at infinite time.

The minimum observation time is determinable from the minimum length of gradient pulses, the subsequent recovery of the apparatus from eddy currents and magneto-acoustic effects and the signal-to-noise ratio.

Long diffusion times are appropriate for our current breast cancer studies. The increase in the average ADC_m_(t_d_) at t_d_ >50 ms, determined from the initial slope of the diffusion decay (Table 3), is explained by the redistribution of the spin populations of both compounds (P_1_ and P_2_), which have different relaxation times T_1_ and T_2_[62]. The ADC_m_ is uninformative in our breast tissue specimens, since it cannot separately estimate the translational mobility of each phase.

In a well-connected porous medium, ADC(t) approaches a non-zero finite value after an extended time. The ADC is reduced by a geometric factor known as the tortuosity, α [65]; specifically, ADC(t) → ∞ → ADC_0_/α. Previous studies [62] have analyzed the long-time behavior in a specific model of packed spherical cells with permeable walls. In this model, the tortuosity factor (α) depends on the permeability. Although α contains geometric information, the same α is obtained in many different geometries [65]. The tortuosity of native breast tissues cannot be evaluated on account because of the tissue complexity. Nevertheless, the ratio (high or low) of the percentage of morphological moieties indirectly reflects the diversity of α within a tissue specimen (Table 1) and also influences the ADC.

### Effects of morphological moieties on the diffusion parameters

Cells aggregate into four major tissue groups: epithelial tissues, supporting and connective tissues (including fatty adipose tissue, cartilage and bone), muscle, and nervous tissue.

Breast contains abundant epithelial tissues and supporting and connective tissues. The latter contain a large amount of extracellular material and ground substance of (mainly) complex carbohydrates and protein polymers. Embryonic fibroblasts differentiate into white and yellow fibers, which form collagen and elastin, respectively. The fibrils of both of these proteins are embedded in the ground substance.

Direct and indirect measurements have proven that NMR parameters are influenced by the biochemical constituents of tissues; that is, their composition and geometrical arrangement (such as morphology and orientation towards a magnetic field) [23, 66, 67].

The mean residence times of free water molecules range from 10^−11^ to 10^−12^ s [68]. In biological media, the mean residence times are < (0.1–1) × 10^−3^ s in tissues [69], (12–25) × 10^−12^ s in ionic solution [70], 1 × 10^−10^ s in lipids [68] and 5 × 10^−9^ s to 1 × 10^−4^ s in proteins [66].

The T_2_ relaxation times of water molecules in collagen gels with magnetically oriented and randomly oriented fibers are 0.52 s and 1.32 s, respectively. The ADCs of water molecules measured with the magnetic pulse gradient parallel and perpendicular to the collagen fibers are 2.08 × 10^−9^ m^2^/s and 1.92 × 10^−9^ m^2^/s, respectively. These differences result from structural changes in the collagen fiber structures induced by the magnetic orientation [66].

Water residence times are also influenced by the secondary structures of sugars (saccharide size, linkage and branching). In particular, they are prolonged, and the translational and rotational dynamics of the water molecules are retarded, in the presence of wide helices and branched sugars. In surrounds of extended helices and smaller oligosaccharides, water dynamics are faster and less hindered. This indicates that the structure and dynamics of carbohydrate surfaces are strongly affected by branching, the type of linkage between monomers, and the anomeric configuration [23].

In nervous tissues, the apparent diffusion coefficient of water is affected by the direction of the axonal fibers [67].

In the present investigation, the self-diffusion coefficients of water in the FDP and SDP restricted or bound with organic and inorganic molecules were evaluated in breast pathology (Tables 2, 4, 5 and 6). Reliable differences were found in the ADC_m_ of tissues excised from all cancer patients, in the D_FDP_ of all tissue specimens and T_2_N_0_M_0_, and in P_1_ in T_2_N_1_M_0_ tissues (Table 2). Correlation studies (Table 4) revealed significant relationships between parenchyma and P_1_ value, stroma percentage and ADC_m_, P_1_ values, and adipose tissue percentage and D_FDP_. The D_SDP_ and P_1_values, pericellular edema percentage and D_SDP_, P_1_ values, vessels and perivascular edemas percentage are not significantly correlated with either of the diffusion parameters.

**Table 4:** **The correlation factors (r) of the parameters of dynamic characteristics of water molecules with the breast tissues’ histological constituents percentage**

Parameter	The group of specimens	Parenchyma	Stroma	Adipose tissue	PCE	PVE	Vessels
ADC_m_	1.1	1.1.1	1.1.2	1.1.3	1.1.4	1.1.5	1.1.6
	Control	−0,497	−0,497	0,671	−0,598	−0,992	0,895
	2.1	2.1.1	2.1.2	2.1.3	2.1.4	2.1.5	2.1.6
	Stroma <50%	0,850	−0,912	−0,876	0,748	−0,837	−0,945
	3.1	3.1.1	3.1.2	3.1.3	3.1.4	3.1.5	3.1.6
	Stroma ≥50%	−0,540	−0,104	0,458	0,823	0,933	0,909
D_FDP_	4.1	4.1.1	4.1.2	4.1.3	4.1.4	4.1.5	4.1.6
	Control	−0,130	−0,291	0,252	−0,080	0,288	−0,186
	5.1	5.1.1	5.1.2	5.1.3	5.1.4	5.1.5	5.1.6
	Stroma <50%	−0,149	0,074	0,260	0,297	0,195	−0,576
	6.1	6.1.1	6.1.2	6.1.3	6.1.4	6.1.5	6.1.6
	Stroma ≥50%	0,841	−0,722	0,500	0,295	−0,756	0,397
P_1_	7.1	7.1.1	7.1.2	7.1.3	7.1.4	7.1.5	7.1.6
	Control	0,701	−0,197	−0,662	0,751	−0,114	−0,413
	8.1	8.1.1	8.1.2	8.1.3	8.1.4	8.1.5	8.1.6
	Stroma <50%	0,678	−0,211	−0,709	0,601	−0,202	0,021
	9.1	9.1.1	9.1.2	9.1.3	9.1.4	9.1.5	9.1.6
	Stroma ≥50%	0,793	−0,895	−0,984	0,800	0,872	−0,998
D_SDP_	10.1	10.1.1	10.1.2	10.1.3	10.1.4	10.1.5	10.1.6
	Control	0,847	−0,130	−0,795	0,763	−0,401	−0,304
	11.1	11.1.1	11.1.2	11.1.3	11.1.4	11.1.5	11.1.6
	Stroma <50%	−0,243	0,093	−0,136	0,239	−0,004	0,106
	12.1	12.1.1	12.1.2	12.1.3	12.1.4	12.1.5	12.1.6
	Stroma ≥50%	0,067	0,256	−0,516	0,979	0,207	−0,611

The significant values of the correlation factors, p < 0,05 :

ADC_m_:1.1- 1.1.2; p=0,041; 2.1-2.1.2; p=0,028; 3.1-3.1.2; p=0,045.

D_FDP_: 5.1 – 5.1.6; p=0,031.

D_SDP_: 10.1 – 10.1.1 p=0,033; 10.1 – 10.1.3 p=0,046; 11.1 – 11.1.3 p=0,028; 12.1 – 12.1.3 p=0,016; P_1_ : 8.1 – 8.1.1; p=0,008; 8.1 – 8.1.3; p=0,004; 8.1 – 8.1.4 p=0,023.

**Table 5:** **Dynamic parameters of breast tissues’ water molecules are influenced by the histological constituents**

Function of dependence	Equation of dependence (p ≤ 0,05)	R ^2^
D_FDP_ = f **(** **P, St, AT, PCE, V)**	D_FDP_ = 4,07^*^-0,06 · **P** ^**1**^-0,09 · **St** + 0,03 · **AT** + 0,08 · **PCE**-0,09 · **V** + 0,001 · **P** ^*^ + 0,001 · **St** ^2^-0,001 · **AT** ^2*^-0,003 · **PCE** ^2^ + 0,005 · **V** ^2^	0,92
D_SDP_ = f **(P, St, AT, PCE, V)**	D_SDP_ = 2,42 + 0,03 · **P**-0,07 · **St**-0,01 · **AT**-0,13 · **PCE** + 0,08 · **V**-0,001 · **P** ^2^ + 0,001 · **St** ^2^ + 0,0002 · **AT** ^2^ + 0,01 · **PCE** ^2^-0,005 · **V** ^2^	0,81
P_1_ = f **(P, St, AT, PCE, V)**	P_1_ = 1,05 + 0,02 · **P**-0,013 · **St**-0,02 · **AT** + 0,07 · **PCE**-0,02 · **V**-0,0004 · **P** ^2^ + 0,00002 · **St** ^2^ + 0,0002 · **AT** ^2^-0,003 · **PCE** ^2^ + 0,004 · **V** ^2^	0,93

Note: ^1^– the value of certain morphological constituents’ percentage, **R** ^**2**^– determination coefficient, *– p ≤ 0,05.

**Table 6:** **Identification of the breast tissues’ nature (malignant, nonmalignant) according to theirs morphological constituents percentage or the dynamical properties of water**

Function of dependence	Equation of dependence (p ≤ 0,05)	R ^2^
CanP^1^ = f **(P, St, AT, PCE, V)**	CanP = −2.18 + 0,099 · **P** ^*^ + 0,031 · **St** − 0,004 · **AT** − 0,02 · **PCE** + 0,04 · **V** − 0,001 · **P** ^2^ − 0,0001 · **St** ^2^ + 0,0004 · **AT** ^2^ + 0,001 · **PCE** ^2^ + 0,0001 · **V** ^2^	0,66
CanP=f**(** **D**_FDP_, **D**_SDP_ **)**	CanP = −3,01 + 8,02 · **D**_FDP_ + 0,84 · **D**_SDP_ − 2,36 · **D**_FDP_^2^ + 0,50 · **D**_SDP_^2^ − 2,78 · **D**_FDP_ · **D**_SDP_	0,69
CanP=f**(** **D**_FDP_, **D**_SDP_,**P**_1_ **)**	CanP = 2,71 + 5,69 · **D**_FDP_ − 0,59 · **D**_SDP_ − 10,21 · **P**_1_ − 1,41 · **D**_FDP_^2^ + 0,70 · **D**_SDP_^2^ + 1,46 · **P**_1_^2^ − 4,19 · **D**_FDP_ · **D**_SDP_ + 4,29 · **D**_FDP_ · **P**_1_ + 2,75 · **D**_SDP_ · **P**_1_	0,85

Note: ^1^– cancer probability (0 → 1); ^*^– the value of certain morphological consituent’s percentage, R^2^– determination coefficient.

Widely variable correlation coefficients, especially those in which the sign depends on the stroma contents, imply a strong influence of the morphological cellular constituents on the tortuosity factor α. This interplay will cause variations in D_FDP_ , D_SDP_ and P_1_.

Even reliable statistical differences (Tables 1 and 2) [19, 21, 37] and high correlations (Table 4) [19] do not imply full causality in NMR studies. If a correlation is largely positive or negative, it is incorrect to conclude that a change in one parameter is solely responsible for a change in the correlated parameter [35]. Conversely, practical regression analysis typically adopts models that are more complex than the first-order (straight-line) model; our research was no exception. The modeling design combined 5 (P, St, AT, PCE, V) assessed morphological constituents. PVE’s percentage depends on the other morphological constituents. The obtained data were fitted to equations that best described the impact of histological compounds on the values of D_FDP_ , D_SDP_ and P_1_ (R^2^ = 0.81 − 0.93, Tables 5 and 6).

From the fittings of D_FDP_ and D_SDP_, P_1_ = f (P, St, AT, PCE, V) (Table 5), we can elucidate the contribution of morphological moieties to the FDP and SDP. Specifically, we find that the non-linear coefficients are 10–50 fold less than the linear ones.

Parenchyma reduces the D_FDP_ because this tissue is rich in endoplasmic reticulum (ER), a 50 − 150-nm-wide complex network of membranes. The rough ER is associated with numerous ribosomes (diameter = 21 − 25 nm). In addition, cells may contain more than 1000 mitochondria; these complex bodies of width 1 μm may occupy almost 24% of the intracellular area [71]. The P_1_ (FDP) was positively influenced by P, which may be partially attributed to the FDP-containing regions of the parenchyma cells (Figure 2B, C).

Stroma located in the extracellular space can significantly reduce the fluid properties of surrounding water molecules by virtue of their collagen fibers, which are embedded in the carbohydrate matrix at inter-fiber distances of 2 μm (Figure 2B). The ADC of regional water negatively correlates with protein concentration [72].

Adipose tissue is essentially lacking in water molecules (Figure 2D). Nevertheless, unexpectedly given its hydrophobic nature, it exerts a positive and negative influence on the D_FDP_ and D_SDP_, respectively.

The effect of adipose tissue on D_FDP_ can be explained by the liquid low-molecular weight ingredients of fats. Short-chain fatty acids (monoolein, ADC =0.1 × 10^−10^ m^2^/s [73]), glycerol and water molecules (ADC =10^−10^ to 10^−9^ m^2^/s) [74] can penetrate the fatty acid’s tails. They also influence the D_SDP_, because the regional water ADC is inversely correlated with the local lipid concentration [72] (Table 4).

Stroma exerts a negative influence in the equations for D_FDP_, D_SDP_ and P_1_ (Table 5). The percentage of vessels in the tissue negatively influences the D_FDP_ and P_1_ and positively influences the D_SDP_ (Table 5). Lumen areas of vessels are characterized by their hematocrit levels and their volume ratios of insoluble/entire blood compounds (≈40%). The negative influence might manifest from the presence of red cells and proteins in the lumen, as well as intramural constituents (elastin) (Figure 2A).

Pericellular edema is a pathological pericellular constituent. This constituent exerts a positive influence on D_FDP_ and P_1_ and a negative influence on D_SDP_.

We find that P, St and PCE depend on the stroma contents of tissues and change sign in the equations describing D_FDP_, D_SDP_, P_1_. These results imply a dual (intracellular and extracellular) origin of both FDP and SDP. We conclude that the dynamic parameters of water in tissues are significantly influenced by the morphological moieties.

### Opportunity for distinguishing tissue malignancy from NMR parameters of water dynamics

The relaxation time of tissues and the dynamic parameters of their contained water depend more heavily on the water content and the extent of necrosis and fibrosis, than on histological structure [75]. Later, tumor cellularity was found to be negatively correlated with mean ADC [19].

Elevated water-fat ratios have been identified in the MR spectra of malignant tissues *in vivo*, and compared with both the normal breast tissue of healthy controls and the contralateral unaffected breast tissue of the patients. When the primary tumor size is reduced by chemotherapy, the water-fat ratio decreases relative to its pre-therapy level [76].

Algorithms that discriminate between benign and malignant breast lesions are divisible into two classes; physiologically model-based and model-free.

Model-based methods focus on the physiological meaning of constructed dynamic-contrast-enhanced (DCE) time curves [77]. These models require additional measurements, such as blood AIF (arterial input function) and pre-contrast T_1_ relaxation rate.

Model-free algorithms attempt to overcome the limitations [77] inherent in diagnostic evaluation of breast cancer. Factor analysis of medical image sequences (FAMIS), principal component analysis (PCA) and independent component analysis reveal the physiological dynamics of the target tissue. Artificial neural network (ANN) is a commonly used clustering algorithm that permits dynamic and textural analysis, and the “fuzzy-c” means (FCM) algorithm incorporates logistic regression texture and age.

In the present study, we verified cancer tissues from their morphology and diffusional parameters (Tables 5 and 6) using non-linear regression analysis [35]. In the linear regression analysis  the determination coefficient was very low (R^2^ = 0.3–0.4).

In all cases, the most relevant factors in cancer diagnosis are the morphological compounds (P, St, AT, V, PCE, and PVE). Inserting these parameters into the equation for cancer probability, namely, (CanP) = f(P, St, AT, PCE, V), cancer was correctly diagnosed in 66% (R^2^ = 0.66) of cases.

In this case, since the non-linear coefficients are 10–400 fold less than the linear ones (CanP = f(P, St…); see Table 5), and the values of (D_FDP_)^2^, (D_SDP_) ^2^, D_FDP_^.^D_SDP_, D_FDP_^.^P_1_ ,D_SDP_^.^P_1_ are much less than 1.0 − (respectively, (10^−9^)^2^ m^2^/s, (10^−11^)^2^ m^2^/s, (10^−9^) m^2^/s, (10^−11^) m^2^/s), P_1_ < 1,0), we can approximate the cancer probabilities by linear functions of D_FDP_, D_SDP_ and P_1_; that is, CanP = f [D_FDP_, D_SDP_) and CanP = f (D_FDP_, D_SDP_, P_1_)] (Table 6).

Cancer probability is positively influenced by the P, St, and V constituents, the main targets in histological cancer revelation. The AT and PCE lack any morphological specificity for classification purposes in cancerous breast tissues, and both parameters exert a negative influence on cancer probability. More observations are required for a definite assessment of these phenomena.

The cancer probability equations defined above are useful because they directly relate the tissue constituents to the dynamical water parameters (D_FDP_, D_SDP_ and P_1_), which are evaluable in *in vivo* studies. The equation CanP = f(D_FDP_, D_SDP_) demonstrates equivalent information efficacy (R^2^ = 0.69) to CanP = f(P, ST, AT). By incorporating P_1_ , R^2^ is significantly increased to 0.85 (Table 6).

**Figure 3:**
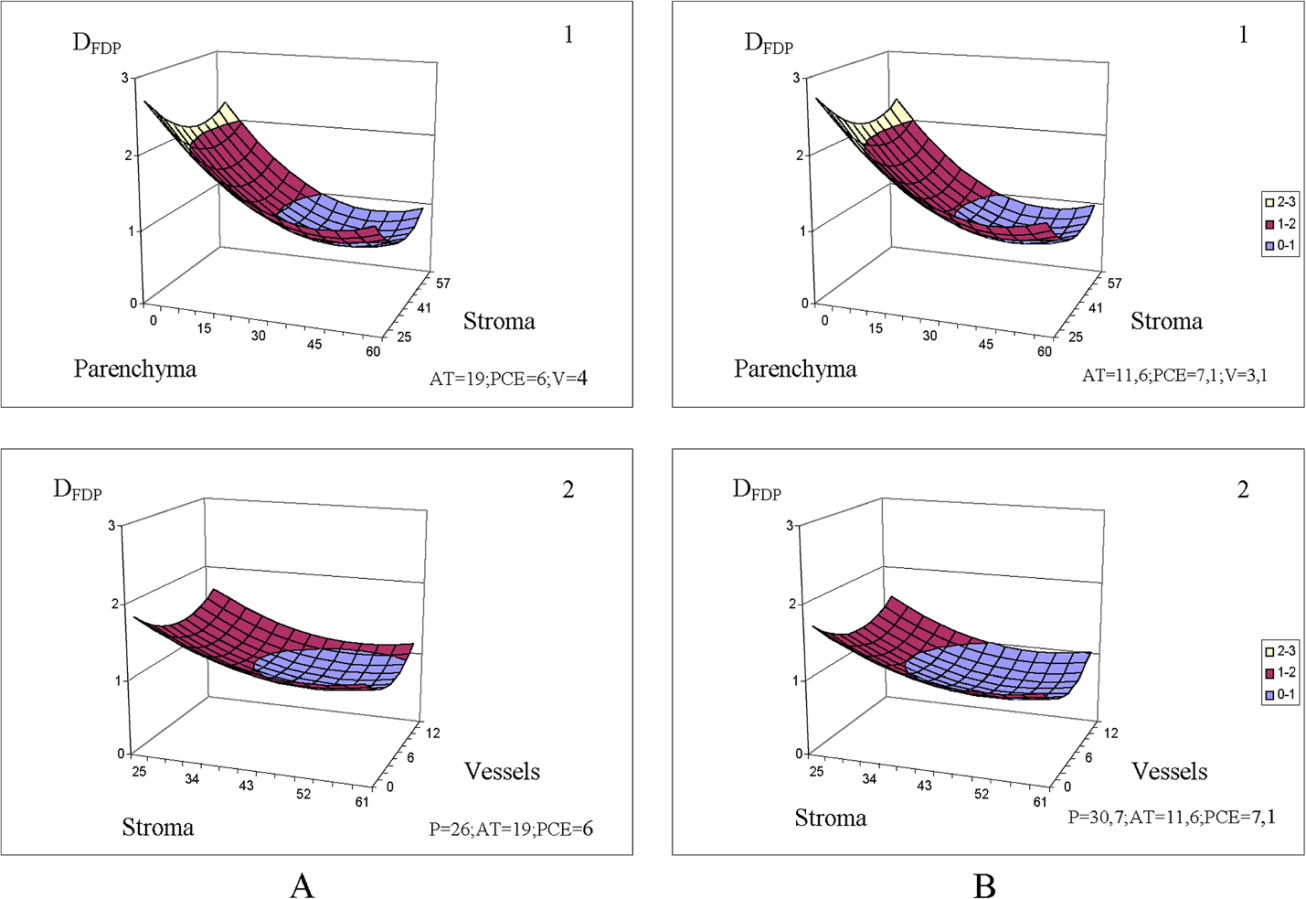
**D**_**FDP**_ **(10** ^**-9**^ **m** ^**2**^ **/s) is stipulated by the morphological constituents’ percentage (%).** The values of fixed parameters (AT, PCE, V, P) were picked as: 1. Mean values of the entire group (control + cancer) of samples, index **A**. 2. Mean values of the malignant specimens only, index **B**. The scale is shared by **A** and **B** section.

**Figure 4:**
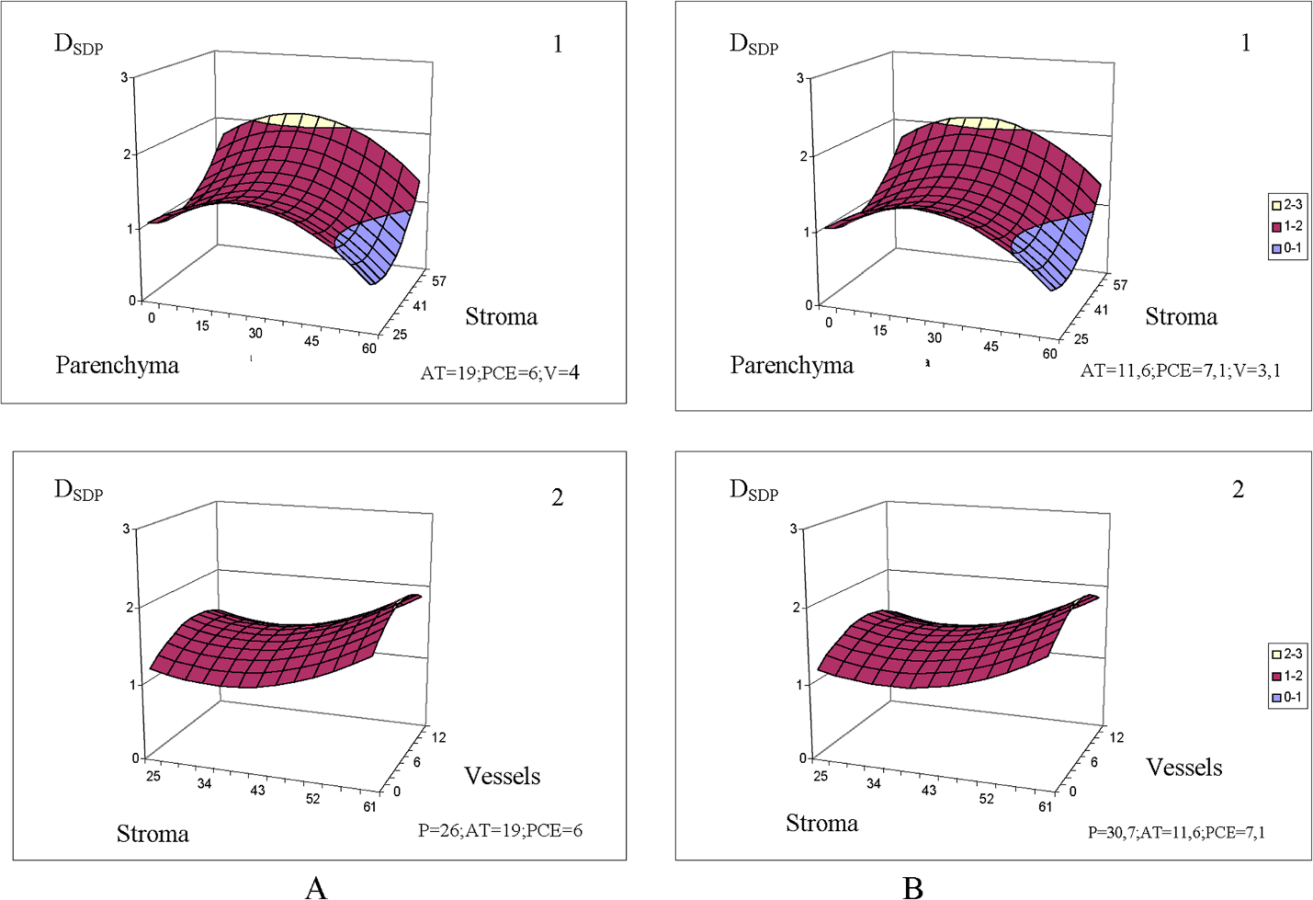
**D**_**SDP**_ **(10** ^**-11**^ **m** ^**2**^ **/s) is stipulated by the morphological constituents, percentage (%).** The values of fixed parameters (AT, PCE, V, P) were picked as: 1. Mean values of the entire group (control + cancer) of samples, index **A**. 2. Mean values of the malignant specimens only, index **B**. The scale is shared by **A** and **B** sections.

**Figure 5:**
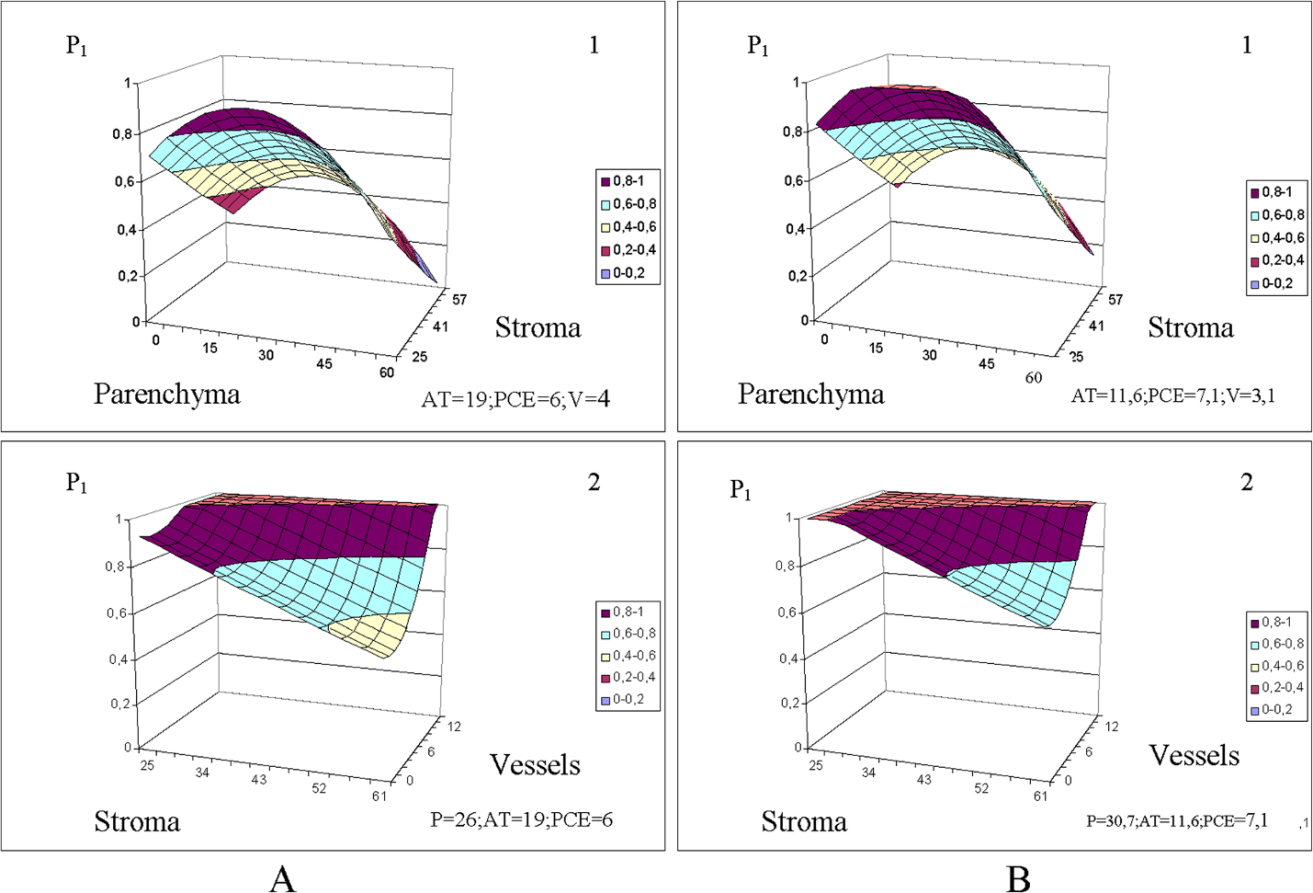
**P**_**1**_ **is stipulated by the morphology constituents, percentage (%).** The values of fixed parameters (AT, PCE, V, P) were picked as: 1. Mean values of the entire group (control + cancer) of samples, index **A**. 2. Mean values of the malignant specimens only, index **B**.

**Figure 6:**
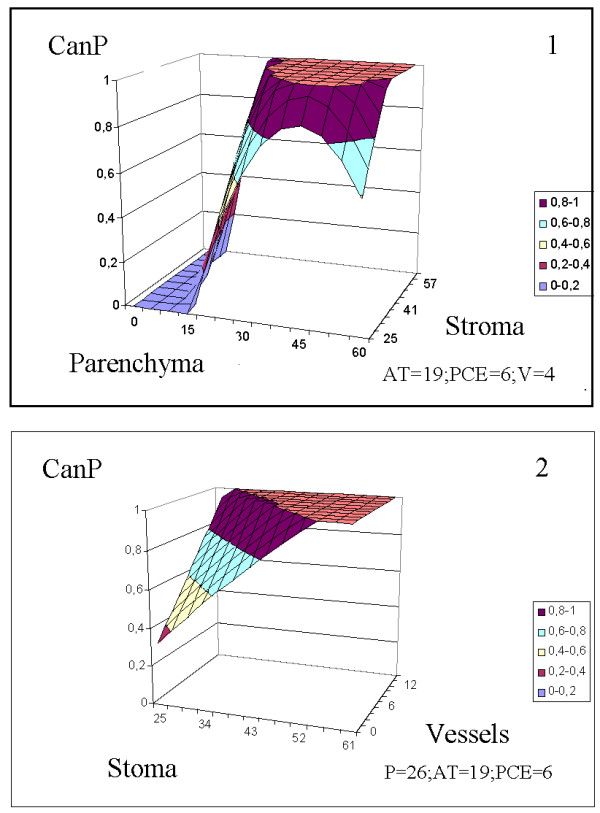
**Cancer tissues identification according to the morphological constituents’ percentage (%).** The values of fixed parameters (AT, PCE, V, P) were picked as mean values of the entire group (control + cancer) of specimens.

**Figure 7:**
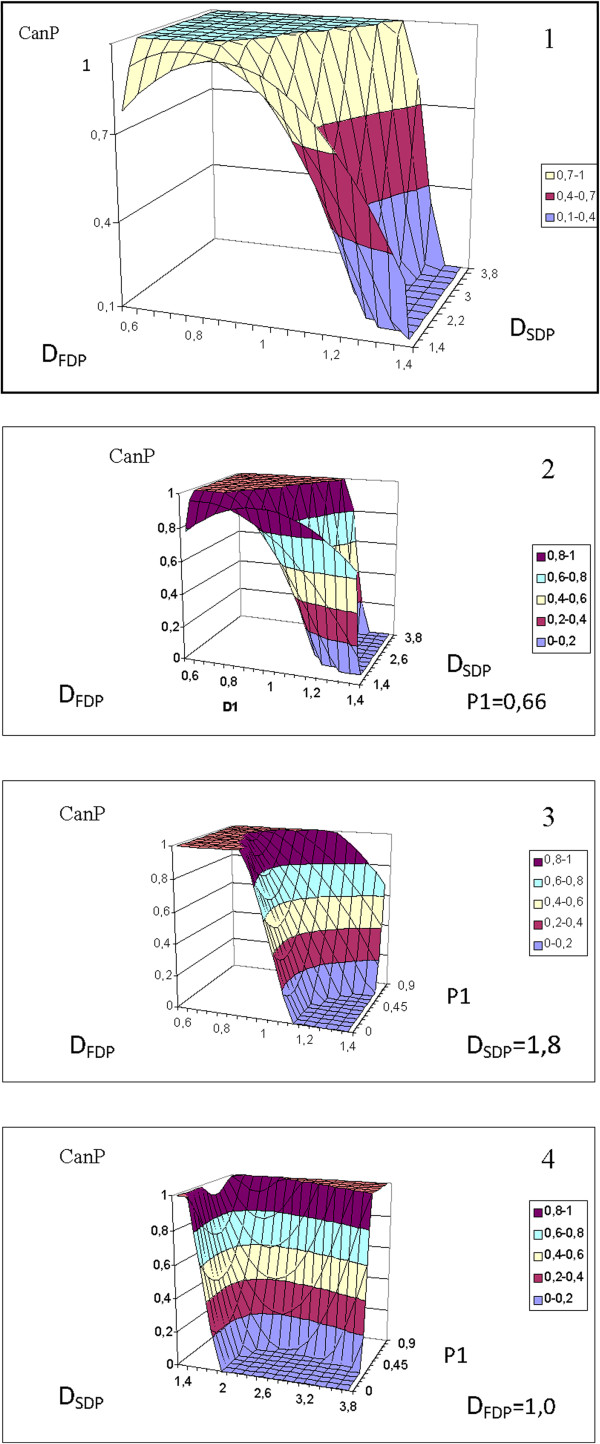
**Cancer tissues identification according to the values of D**_**FDP**_ **10** ^**-9**^ **m** ^**2**^ **/s, D**_**SDP**_ **10** ^**-11**^ **m** ^**2**^ **/s (1), D**_**FDP**_ **10** ^**-9**^ **m** ^**2**^ **/s, D**_**SDP**_ **10** ^**-11**^ **m** ^**2**^ **/s, P**_**1**_ **(2-4).** The values of fixed parameters (D_FDP_, D_SDP_, P_1_) were picked as mean values of the entire group (control + cancer) of specimens.

The predictions of the equations are visually clarified by the 3D plots in Figures 3, 4, 5, 6 and 7. The fixed parameters (AT, PCE, P, V) input to the appropriate equations (Table 5, Figures 3, 4 and 5) were selected as (1) the mean values of the entire sample group (control + cancer; Figures 3, 4 and 5, panels **A**) and (2) the mean values of the malignant samples only (Figures 3, 4 and 5, panels **B**).

P, St and V were selected as the parameters for which D_FDP_, D_SDP_ and P_1_ depend on (1) the proportion of parenchyma, which reflects the cellularity of the tissue; (2) the percentage of stroma (collagen), which influences the ADC [66] (Table 4, ADC_m_), and whose biosynthesis is altered in cancer tissue [78], and (3) micro-vessel counts, which are higher in malignant than in benign pathologies [79, 80].

The influences of the remaining constituents, PVE, PCE [81] and especially AT[82], are of immense interest, but a detailed analysis of D_FDP_, D_SDP_ and P_1_ as functions of the cell histology is beyond the scope of this paper. Nevertheless, we can highlight two features: 1. We can observe the sensitivity of the equations (Table 6) D_FDP_ = f (P, St, AT, PCE, V), D_SDP_ = f (P, St, AT, PCE, V) P_1_ = f (P, St, AT, PCE, V) to fluctuations in the morphological constituents of the specimen tissues. Sensitivity analyses can be conducted over a wide range of parenchyma percentages (0 − 60%), stroma percentages (25 − 61%), and vessel percentages (0 − 12%), considering both the entire group of specimens [Figures 3A(1–2), 4A(1–2), 5A(1–2)] and malignant specimens only [Figures 3B(1–2), 4B(1–2), 5B(1–2)].

2 Because the plots of CanP(St, P) and CanP(St, V) in Figure 6, and those of CanP (D_FDP_, D_SDP_) and CanP (D_FDP_, D_SDP_, P_1_) in Figure 7 develop flat regions at 0 and 1, we can identify tissue specimens that are unambiguously malignant (**1**) or certainly nonmalignant (**0**).

The obtained equations avoid the need for procedures that are essential in DCE–MRI [77]; namely, intravenous contrast injection, division of patient data into training and test datasets, high computational cost in image processing, and accentuation of the breast cancer region by an expert.

## Conclusion

Biological tissue comprises 65 − 75% water. Because the concentration of pure water is 55 Mol (^1^H concentration =110 Mol), the NMR signal is detectable even in tiny voxels, where the tissue extends by <0.5 mm each side.

The number of water molecules exhibiting free and hindered diffusion may differ among the various compartments of a tissue. The intrinsic ADCs may also vary among these compartments. Furthermore, a certain proportion of the water molecules in each compartment may be restricted by impermeable and semipermeable barriers, depending on the tissue morphology (biochemical composition, geometry and size of the confining compartment), the diffusion coefficients, and the time over which the diffusion process is probed. The ADC reflects the compartmentalization of water more by its dynamic properties than by its histological location.

Histological components of the tissue are related to the diffusion biexponential model parameters. Therefore, they can be used to determine the relative probability of cancer in a given specimen with some certainty.

## Authors’ information

RF, Full professor of Biochemistry, МD, PhD, Dr. Sci. Med. ; RA, MD; KK, MD; SZ, MD, PhD; FR, biologist; TA, PhD.

## Abbreviations

n: Number of samples
FDP: Fast diffusion phase of water
SDP: Slow diffusion phase of water
ADC: Apparent diffusion coefficient
ADC_m_: Averaged apparent diffusion coefficient
P: Parenchyma
St: Stroma
AT: Adipose tissue
V: Vessels
PCE: Pericellular edema
PVE: Perivascular edema
PFG: Pulsed field gradient
DW-MRI: Diffusion weighted MRI.
